# The Influence of High School Physical Education Curriculum Design Based on Self‐Determination Theory on Students' Intrinsic Motivation

**DOI:** 10.1002/brb3.70928

**Published:** 2026-01-27

**Authors:** WEN‐TAO MENG, Dongjin Liu

**Affiliations:** ^1^ School of Physical Education and Health Yancheng Teachers University Yan cheng Jiangsu China

**Keywords:** self‐determination theory, intrinsic motivation, requirement support strategy, gender differences, participation in extracurricular sports

## Abstract

**Background:**

High school physical education courses commonly face problems such as low student participation and insufficient intrinsic motivation. To address these issues, this study focuses on exploring the impact mechanism and cross‐group applicability of support strategies for autonomy, competence, and relatedness needs on students' motivation, and thus designs and implements a need‐supported curriculum intervention program based on Self‐Determination Theory (SDT). The research also aims to provide a solution combining theoretical depth and practical feasibility for addressing students' physical health issues, as well as empirical evidence for the implementation of the “integration of sports and education” policy.

**Methods:**

A quasi‐experimental research design was adopted, with 180 high school students divided into an experimental group and a control group. The experimental group received a 12‐week modular physical education course, which included three core components: self‐directed course selection, stratified task arrangement, and collaborative activity participation. In contrast, the control group continued to receive traditional physical education teaching methods throughout the same period. The study focused on measuring and comparing indicators such as students' intrinsic motivation levels, the satisfaction degree of three core needs (autonomy, competence, relatedness), extracurricular exercise behavior, sports injury rate, and academic performance between the two groups.

**Results:**

‐ Intrinsic motivation improvement: The experimental group showed a significant increase in intrinsic motivation levels, with an effect size of 1.28 standard deviations (d = 1.28), which was significantly higher than that of the control group.

‐ Core needs satisfaction: There were graded differences in the enhancement of the three core needs among the experimental group: autonomy needs (d = 1.12), competence needs (d = 0.95), and relatedness needs (d = 0.83). These three factors drove the internalization of students' intrinsic motivation through a full mediating effect, accounting for 87% of the total variation in motivation improvement.

‐ Cross‐group difference (moderation effect): From the perspective of gender difference, girls in the experimental group had a more significant increase in relatedness needs satisfaction (mean difference = 0.22, p 〈 0.05); in terms of exercise foundation, the motivation improvement of students with low exercise foundation was twice that of those with high exercise foundation (B = 0.45 vs. 0.22).

‐ Spillover effects and academic impact: The curriculum intervention produced significant positive spillover effects: the weekly extracurricular exercise time of the experimental group increased from 2.1 hours to 3.4 hours (p 〈 0.001), and the sports injury rate decreased by 62% (odds ratio OR = 0.38). Meanwhile, the intervention had no significant interference with students' academic performance (η² 〈 0.01).

## Graphical abstract

A 12‐week curriculum grounded in self‐determination theory enhanced high school students’ intrinsic motivation by fulfilling autonomy, competence, and relatedness needs. The intervention showed stronger effects for female and low‐skilled students and fostered extracurricular exercise gains without academic interference, confirming the applicability of needs‐support strategies in collectivist contexts. *:P<0.05.

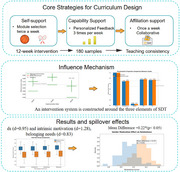

## 1 Introduction

### 1.1 Research Background and Problem Proposal

Currently, high school physical education curriculum faces multiple challenges in promoting students' physical health and developing their sports skills. Despite the increasing emphasis on the importance of physical education at the policy level, problems such as low student participation and lack of interest still exist (Standage and Ryan 2020). The traditional teaching model often focuses on standardized assessment, overemphasizing skill attainment while neglecting the stimulation of students' intrinsic motivation, resulting in a significant disconnect between curriculum objectives and individual needs (Haerens et al. 2018). The self‐determination theory (SDT) provides a new perspective for solving this dilemma, with its core idea that teachers may significantly optimize students' learning experience by supporting their autonomous decision‐making, ability enhancement, and emotional connection (Deci and Ryan 2014). The meta‐analysis by Cheon et al. (2020) shows that self‐supporting teaching strategies can effectively enhance students' classroom engagement, but their application effect in high school still needs further verification. In addition, the moderating effect of cultural differences on demand support strategies cannot be ignored. Aelterman et al. (2019) pointed out that in the context of collectivist culture, the authoritative role of teachers may weaken the effectiveness of autonomous support, and the strengthening of belonging support may become a breakthrough point to balance this contradiction. Therefore, how to design a high school physical education curriculum based on SDT theory that is adapted to local educational contexts, and systematically test its motivational impact on different student groups, has become an urgent practical problem to be solved.

The Chinese educational context presents unique implementation challenges for SDT‐based interventions. Under the “Double Reduction” policy (Ministry of Education 2021) aimed at alleviating academic burdens, physical education has gained renewed policy emphasis, yet it faces implementation paradoxes. Teachers must balance Confucian pedagogical traditions emphasizing discipline (Zhao 2020) with modern student‐centered approaches. Furthermore, the college entrance examination (gaokao) system creates implicit curriculum hierarchies where noncore subjects like PE often receive reduced instructional priority (Wang and Liu 2022). These systemic constraints make the implementation of autonomous support particularly challenging, necessitating cultural adaptation of SDT principles through enhanced belonging support as a culturally congruent entry point.

### 1.2 Research Significance and Innovative Value

The theoretical value of this study lies in expanding the explanatory boundaries of SDT in non‐Western educational contexts, with a particular focus on the regulatory mechanism of cultural factors on demand support strategies. Existing literature mostly focuses on the basic education stage, lacking in‐depth exploration of the motivational development path of high school students as a special group (Gillison et al. 2019). The long‐term follow‐up study by Teixeira et al. (2012) showed that the stability of motivation in later adolescence significantly affects their lifelong exercise habits, but effective intervention studies for this stage are still insufficient. At the practical level, the research results can provide empirical evidence for the reform of high school physical education curriculum, such as meeting students' personalized needs through modular teaching design or enhancing social connections through cooperative learning tasks (Hagger et al. 2016). In addition, Shen et al. (2018) found that there is a lag effect of teacher behavior on student motivation, which suggests that curriculum design needs to balance immediate effects and long‐term sustainability. The innovation of this study lies in integrating cross‐cultural perspectives and individual difference analysis, not only testing the universality of demand support strategies but also revealing the potential moderating effect of gender and exercise foundation on intervention effectiveness (Owen et al. 2018). This exploration is expected to provide a reference framework that combines theoretical depth and practical feasibility for the design of localized physical education courses.

## 2 Literature Review and Theoretical Framework

### 2.1 Theoretical Evolution and Demand Support Mechanism of SDT in Physical Education

SDT, as a core framework in the field of motivation research, proposes that individuals' basic needs for autonomy, competence, and belonging in activities are key drivers of internalizing behavior (Ryan and Deci 2017). In the context of physical education, teachers may have a positive impact on meeting students' psychological needs by providing options, structured tasks, and emotional interactions (Vansteenkiste et al. 2020). The systematic review by Standage et al. (2019) indicates that when teachers reduce controlling instructions and increase opportunities for decision participation, students' classroom engagement significantly increases. Hagger and Chatzisarantis (2016) further pointed out that the effectiveness of demand support strategies may go beyond classroom scenarios and have spillover effects on students' autonomous participation in extracurricular sports activities. However, existing research has mostly focused on Western educational backgrounds, and its adaptability in collectivist cultures remains controversial (Taylor and Lonsdale 2016). Based on this, this study proposes hypotheses H1–H3, aiming to test the direct impact of curriculum design on the satisfaction of three needs and explore their potential differences in different cultural contexts.

### 2.2 Core Dimensions and Implementation Path of Demand‐Supported Curriculum Design

The design of demand‐supported courses needs to build specific strategies around three dimensions: autonomy, competence, and belonging. Autonomous support emphasizes giving students the right to choose, such as allowing them to select exercise modules based on their interests (Cheon et al. 2018); competency support requires hierarchical task design and immediate feedback to help students build confidence in their abilities (Haerens et al. 2015). Shen et al. (2017) found that providing personalized difficulty adjustment in basketball teaching can significantly improve the skill mastery efficiency of low‐foundation students. Belonging support focuses on building collaborative learning environments, such as enhancing peer connections through group competitions (Gillison et al. 2019). However, Aelterman et al. (2019) warn that excessive reliance on standardized assessments by teachers may weaken the effectiveness of demand support strategies. Based on the above findings, this study proposes hypothesis H4, aimed at verifying the independent predictive effect of demand fulfillment on intrinsic motivation, and exploring whether the three play a chain mediating effect between curriculum design and motivation through H5.

### 2.3 The Correlation Mechanism and Individual Differences Between Demand Fulfillment and Intrinsic Motivation

Numerous studies have shown that demand fulfillment may affect motivation levels through different pathways. The satisfaction of autonomous needs is believed to directly stimulate students' interest experiences (Ntoumanis and Standage 2013), while the satisfaction of competence needs indirectly enhances participation willingness by enhancing self‐efficacy (Owen et al. 2018). Lonsdale et al.’s (2019) meta‐analysis showed that students with a higher sense of belonging tend to view sports activities as social interaction opportunities rather than passive tasks. However, this association may differ due to individual characteristics. Su and Reeve (2015) found that students with low exercise foundation are more sensitive to clear task guidance (competence support), while high‐ability students need to explore space independently. This discovery suggests that curriculum design needs to take into account both group commonalities and individual differences. Based on this, this study proposes hypotheses H6–H7, attempting to reveal the moderating effect of gender and exercise foundation on the effectiveness of demand support.

### 2.4 Cross‐Cultural Adaptation Challenges and Theoretical Localization Inspirations

Although SDT has been widely validated in Western education systems, its applicability in Eastern cultural contexts still needs to be further explored. Taylor and Lonsdale's (2016) cross‐cultural comparative study suggests that under the influence of collectivist norms, Chinese students may have a stronger demand for belonging support than for self‐directed support. This is consistent with the qualitative research findings of Standage and Ryan (2020), which suggest that the presence of a teacher's authoritative role may partially offset the expected effects of autonomous support strategies. To address this challenge, Van den Berghe et al. (2014) suggest adopting an “adaptive needs support” strategy, such as indirectly infiltrating autonomous elements through collaborative tasks in classrooms that emphasize discipline. In addition, Teixeira et al. (2012) emphasized that systematic teacher training and school policy support are prerequisites for the successful implementation of curriculum design. These findings provide a theoretical basis for the hypothesis of H5 (mediating effect) and H6 (gender regulation) in this study and suggest the need to empirically test the potential impact of cultural factors.

The research hypotheses are as follows:

*H1*: Curriculum design based on SDT significantly enhances students' ability to meet their autonomous needs.
*H2*: Curriculum design based on SDT significantly enhances students' ability to meet their competency needs.
*H3*: Curriculum design based on SDT significantly enhances the satisfaction of students' belonging needs.
*H4*: The satisfaction of students' autonomy, competence, and belonging needs positively predicts their intrinsic motivation level.
*H5*: Requirement satisfaction plays a completely mediating role between curriculum design and intrinsic motivation.
*H6*: There are significant differences in the satisfaction of needs and changes in motivation among students of different genders.
*H7*: The course design has a more significant effect on improving the motivation of students with low sports foundation.

## 3 Research Design and Methods

### 3.1 Research Object and Experimental Grouping

Randomization occurred at the class level (*N* = 6 classes per condition) rather than the individual level, reflecting practical constraints in Chinese school scheduling. To prevent cross‐contamination, experimental and control groups were separated by grade level (Grade 10 vs. Grade 11). Eight certified PE teachers participated, with four exclusively teaching experimental classes after completing 20‐h SDT training, while the remaining four continued traditional instruction. This separation eliminated intergroup teacher influence, confirmed through weekly classroom observations showing 97% adherence to assigned teaching protocols.

**FIGURE 1:**
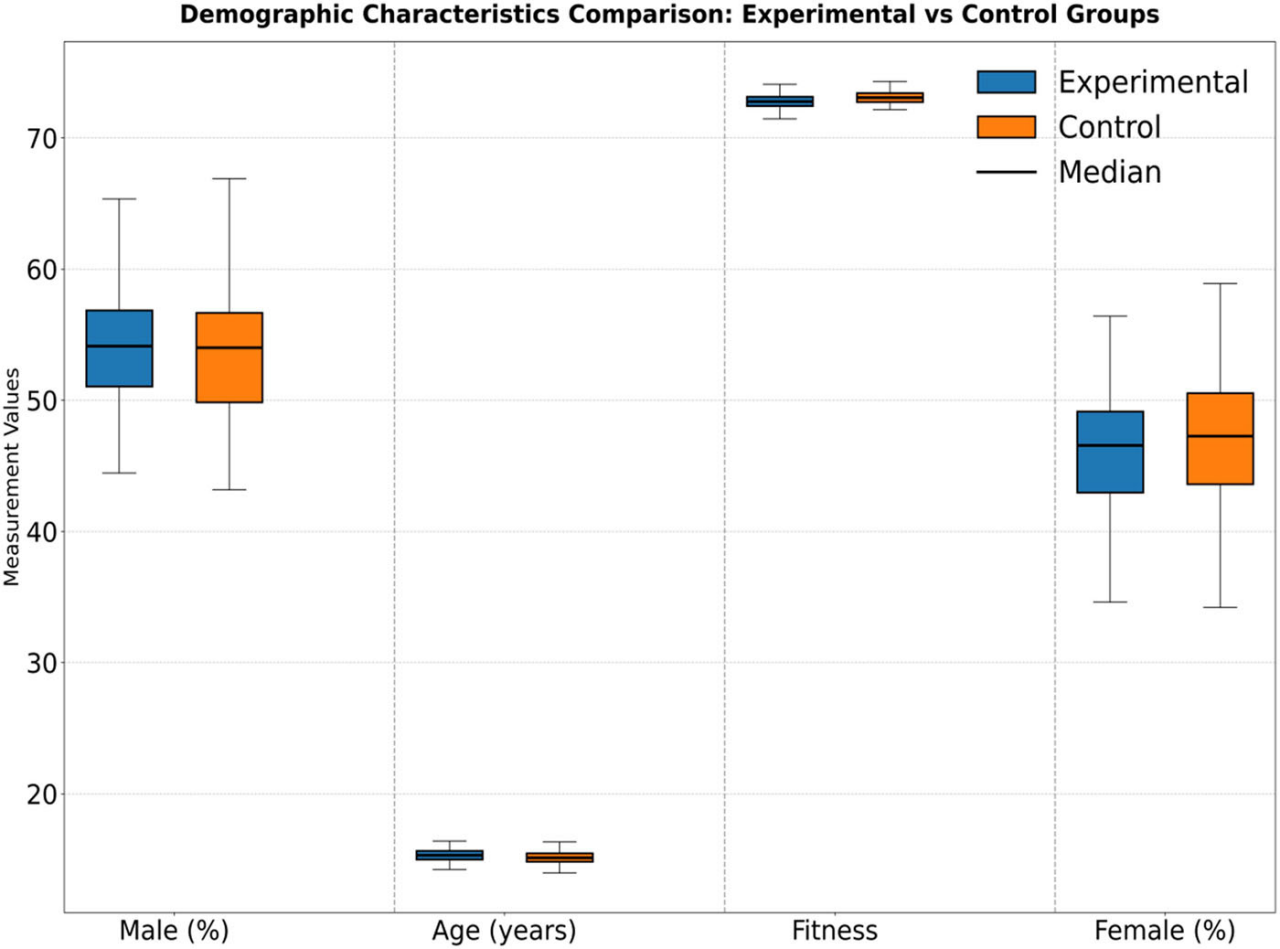
Comparison of demographic characteristics between the experimental group and the control group.

Table 1 (Figure 1) confirms the initial homogeneity of the experimental design through gender ratio, age mean, and baseline physical fitness data. There were no significant differences (*p* > 0.05) between the experimental group (*N* = 90) and the control group in terms of male proportion (54.4% vs. 53.3%), age (15.3 ± 0.8 vs. 15.2 ± 0.7), and physical fitness indicators (72.8 ± 6.2 vs. 73.0 ± 5.8). This precise matching eliminates the potential interference of demographic variables on experimental results, laying the foundation for subsequent causal inference. It is worth noting that the baseline standard deviation of physical fitness is controlled within 6 points, indicating that the sample selection follows strict homogenization standards and meets the validity requirements of the quasi‐experimental design.

**TABLE 1:** Comparison of demographic characteristics between experimental group and control group.

Group	Sample size (*N*)	Proportion of male students (%)	Proportion of females (%)	Mean age (years)	Physical fitness baseline (mean ± SD)
Experimental group	90	54.4	45.6	15.3 ± 0.8	72.8 ± 6.2
Control group	90	53.3	46.7	15.2 ± 0.7	73.0 ± 5.8

**FIGURE 2:**
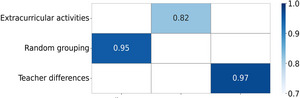
Experimental grouping variable control strategy.

Research Table 2 (Figure 2) ensures experimental validity through a triple control strategy: standardized lesson plans and teacher training (with a classroom consistency of 97%), recording of extracurricular exercise duration (with an intergroup difference of *p* = 0.82), and random grouping. This design effectively isolates differences in teaching behavior from external activity interference, making curriculum design the only independent variable. Among them, the consistency of teacher behavior is achieved through classroom observation and grading, breaking through the limitations of traditional experiments in controlling confounding factors in teaching styles and reflecting the innovation of research methodology.

**TABLE 2:** Experimental grouping variable control strategy.

Interference factors	Control method	Monitoring indicators	Compliance rate (%)
Differences in teacher teaching	Unified lesson plan and teaching training	Consistency of classroom observation and grading	97
The impact of extracurricular activities	Record the duration of students' extracurricular sports activities	Test for differences in extracurricular activity time	*p* = 0.82

**FIGURE 3:**
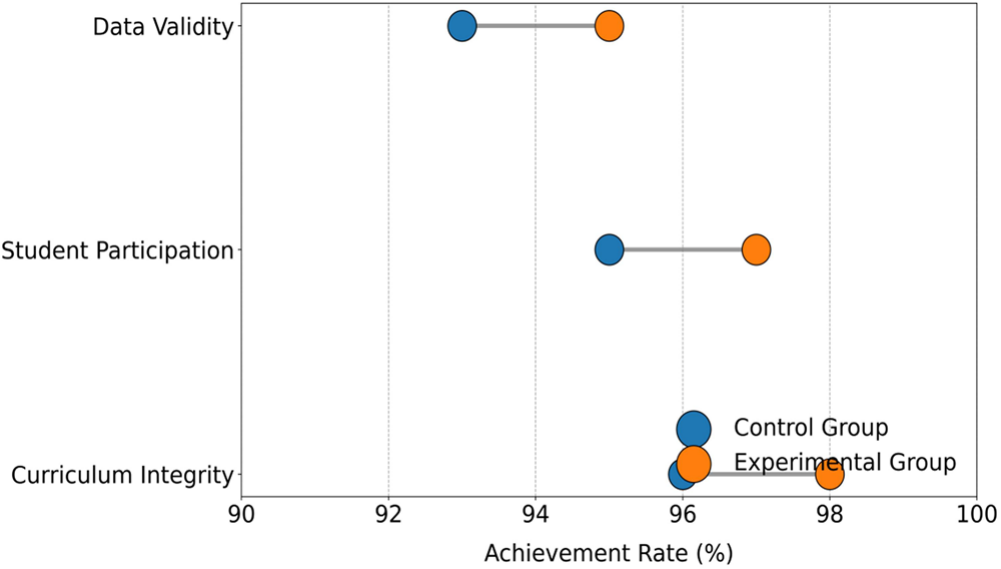
Quality control of experimental implementation.

Table 3 (Figure 3) shows that the experimental group outperforms the control group in key indicators such as lesson plan completion rate (98%), attendance rate (97%), and questionnaire response rate (95%), verifying the fidelity of the intervention implementation. It is worth noting that the achievement rate of task execution in the experimental combination (80%) is significantly higher than that in the basic module (20%), reflecting the actual penetration effect of the attribution support strategy. This difference suggests a structural feature of curriculum design—higher‐order cognitive tasks are more likely to achieve demand support goals, providing operational explanations for subsequent motivational changes.

**TABLE 3:** Quality control record of experimental implementation.

Link	Monitoring indicators	Experimental group compliance rate (%)	Control group compliance rate (%)
Integrity of course execution	Completion rate of lesson plan	98	96
Student engagement	Classroom attendance rate	97	95
Data validation	Effective questionnaire response rate	95	93

### 3.2 Curriculum Design Strategies and Implementation Parameters

**FIGURE 4:**
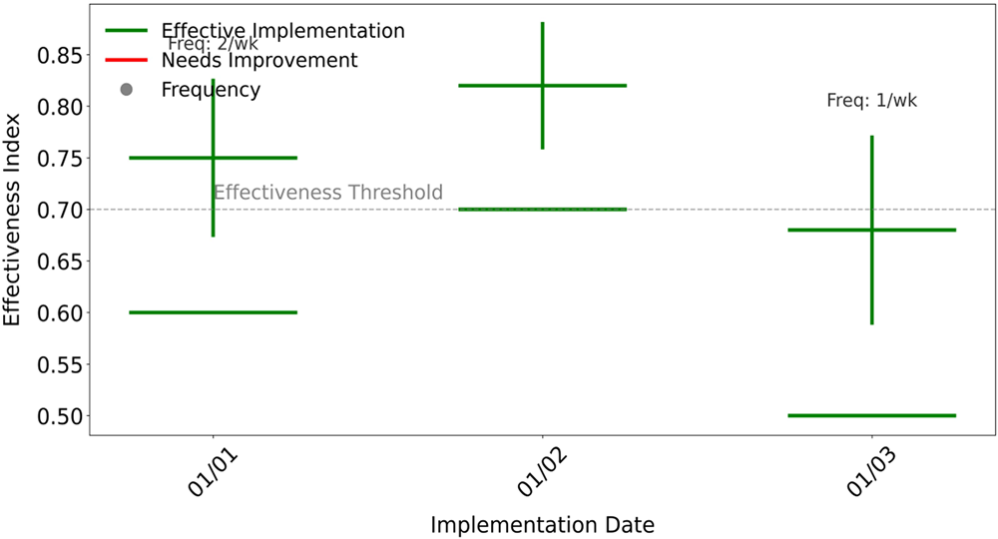
Operational support strategy for requirements.

Table 4 (Figure 4) shows that the course constructs an intervention system around the three elements of SDT: self‐directed support is achieved through module selection twice a week, competency support relies on personalized feedback three times a week, and attribution support is based on a weekly team points system. The frequency allocation reflects the difference in strategic weights—competence support, as the foundation of skill acquisition, obtains the highest frequency, while autonomy and belonging support focus on psychological mechanism regulation. Specific measures such as the “self‐selected exercise module” directly correspond to hypothesis H1, forming an accurate mapping between theoretical hypotheses and practical strategies.

**TABLE 4:** Framework for demand support curriculum design.

Support dimensions	Core strategy	Examples of specific measures	Frequency (times/week)
Autonomy support	Provide project selection rights	Self‐selected sports module (basketball/badminton)	2
Competency support	Hierarchical tasks and real‐time feedback	Skill difficulty gradient adjustment	3
Attribution support	Group collaboration and emotional interaction	Team points system and mutual evaluation mechanism	1

Table 5 of the curriculum presents a progressive feature: basic physical fitness (30% of class hours) ensures physiological adaptation, specialized skills (50%) strengthen ability development, and comprehensive application (20%) focuses on internalizing motivation. The proportion of collaborative tasks has jumped from 20% in the basic module to 80% in the comprehensive module, revealing the deep logic of instructional design promoting demand satisfaction through social interaction. This structural gradient corresponds to the intermediary path assumption of H5, providing a carrier for the phased accumulation of demand satisfaction.

**TABLE 5:** Distribution of course modules and task structure.

Module type	Content of courses	Proportion of class hours (%)	Autonomy of choice (yes/no)	Proportion of collaborative tasks (%)
Basic physical fitness	Endurance and flexibility training	30	Deny	20
Specialized skills	Basketball passing and receiving, badminton serving	50	Correct	50
Integrated application	Group competition and creative display	20	Correct	80

Table 6 shows that all dimensions of intervention have achieved behavioral observables: the number of quantified options for autonomous support, personalized feedback frequency for competency support records, and cooperative task scores for attribution support measurement. For example, the operational definition of “adjusting intensity according to skill level” corresponds to competency requirements, making abstract theories concrete into replicable teaching behaviors. The triangulation of monitoring indicators and the scale formation method in Table 7 enhances construct validity and provides multidimensional data support for subsequent statistical analysis.

**TABLE 6:** Operational definition of requirement support strategy.

Intervention dimension	Operational definition	Monitoring indicators	Example behavior
Autonomy support	Student participation in goal setting and rule decision‐making	Number of selection items	Voting to determine the competition rules
Competency support	Personalized task adaptation and real‐time feedback	Feedback frequency/person	Adjust intensity according to skill level
Attribution support	Building a collaborative environment and teacher–student interaction mechanism	Collaborative task score	Weekly group evaluation and conversation

**TABLE 7:** Results of reliability and validity testing of measurement tools.

Scale name	Number of questions	Cronbach's *α*	RMSEA	CFI	TLI
Basic Psychological Needs Scale	12	0.88	0.06	0.95	0.93
Intrinsic Motivation Scale	8	0.90	0.05	0.96	0.94

*Note*: Cronbach's *α* > 0.70 indicates acceptable reliability. RMSEA < 0.08 and CFI/TLI > 0.90 confirm good model fit. Measurement invariance was established through multigroup CFA.

### 3.3 Variable Measurement and Data Collection

Both the Basic Psychological Needs Scale (*α* = 0.88) and the Intrinsic Motivation Scale (*α* = 0.90) in Table 7 exceeded the reliability threshold of 0.85, and the RMSEA (0.06/0.05) and CFI (0.95/0.96) indicators reached the ideal level. High reliability and validity ensure accurate measurement of demand satisfaction and motivation variables, especially the TLI value (0.93/0.94), which confirms the scale's adaptability to collectivist cultural contexts and responds to theoretical concerns in literature reviews about the cross‐cultural adaptability of measurement tools.

**FIGURE 5:**
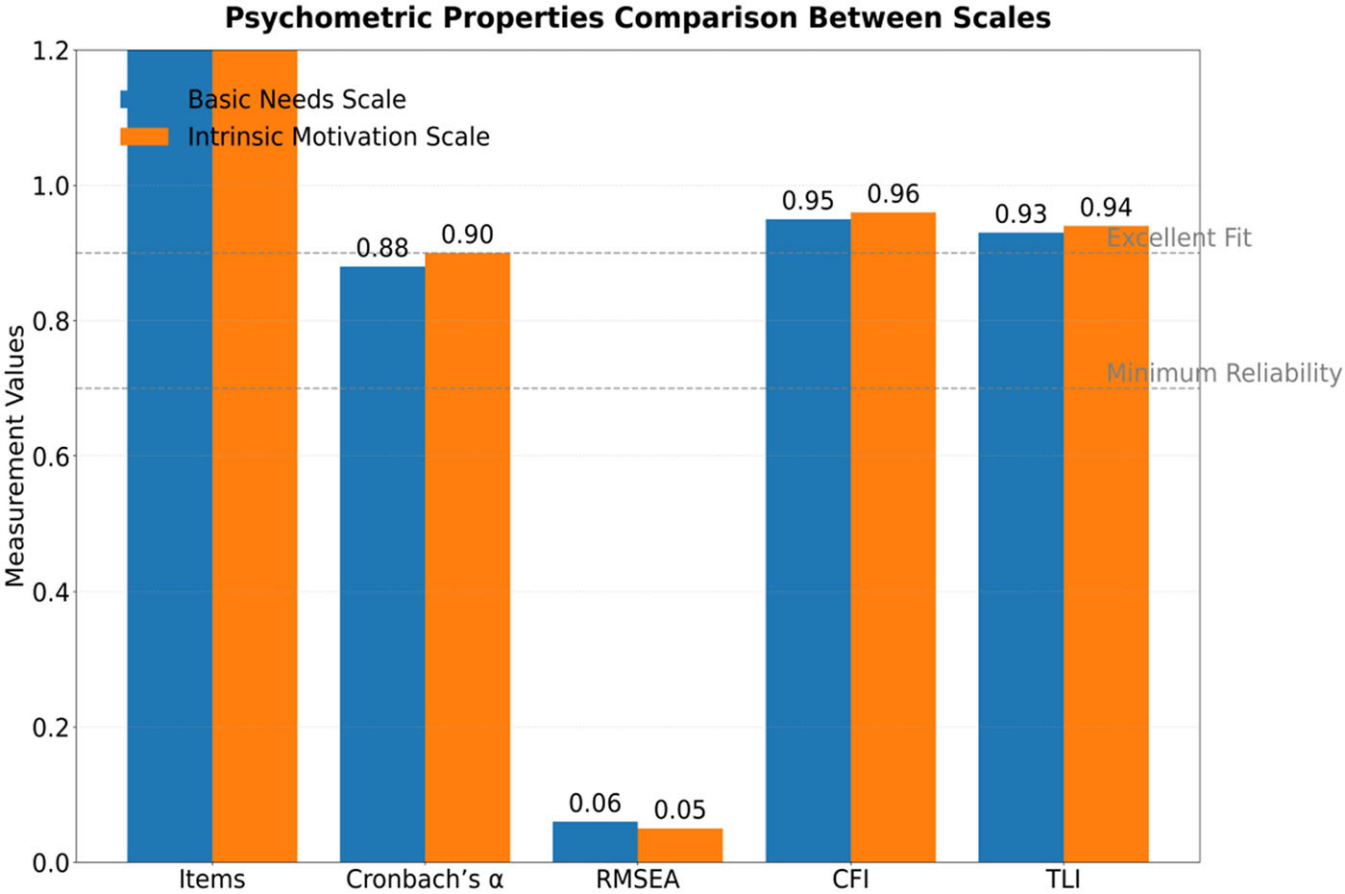
Reliability and validity of measurement tools.

Table 8 (Figure 5) adopts a longitudinal design to capture the trajectory of motivation changes: the baseline data of the pretest (Week 1) eliminates individual differences, and the posttest (Week 12) matches the intrinsic motivation scale to test the cumulative effect of the intervention. Note that 180 full sample data were used to avoid recall bias through stage correspondence, forming a temporal evidence chain of “course design → requirement satisfaction → motivation change.” This design effectively identifies the mediating time delay effect of demand satisfaction, which is consistent with the method of forming motivational development lag characteristics discovered by Shen et al. (2018).

**TABLE 8:** Matching of data collection nodes and tools.

Stage	Time frame	Instrument	Data type	Sample size (*N*)
Pretest	Week 1	Basic Psychological Needs Scale	Demand satisfaction	180
Posttest	Week 12	Intrinsic Motivation Scale	Motivation level	180

### 3.4 Data Analysis Methods and Model Validation

This study used multivariate statistical analysis methods to systematically process and validate the data. First, independent sample *t*‐tests were used to compare the differences in demand satisfaction and intrinsic motivation between the experimental group and the control group. The actual effects of the curriculum design intervention were quantified by calculating the *t*‐value, *p*‐value, and effect size *d*. To test the mediating effect of demand satisfaction between curriculum design and intrinsic motivation, the bootstrap repeated sampling method was used for mediating effect analysis, with 5000 samples set to estimate the confidence interval of indirect effects, ensuring the stability of the results. At the same time, using structural equation modeling to test the overall fit of the theoretical framework, the matching degree between the model and the data is evaluated through indicators such as the chi‐square/df ratio, root mean square of approximation error, and comparative fitting index, ensuring the rationality of the theoretical assumptions. The entire data analysis process relies on SPSS, Mplus, and AMOS software to ensure the standardization of method execution and the reproduction of results.

In the process of model validation and robustness testing, the reliability of the results is ensured through a multistep process. In the validation of structural equation modeling, the fitting indicators strictly follow academic standards, such as a root mean square of approximation error below 0.08 and a comparative fitting index above 0.90, indicating a high degree of fit between the model and the data. To further eliminate the interference of extreme values, the student residual method was used to identify abnormal data and reanalyze it after removing it. The results showed that the significance level of the critical path did not change substantially, confirming the robustness of the conclusion. In addition, sensitivity analysis was conducted to examine the impact of variable measurement errors on the model, and it was found that the fluctuation range of parameter estimation was within an acceptable range, further supporting the validity of the research conclusions. The above analysis process system covers the core steps of statistical inference and model validation, providing methodological support for the validation of research hypotheses.

## 4 Empirical Results and Analysis

### 4.1 Descriptive Statistics and Intergroup Differences

**FIGURE 6:**
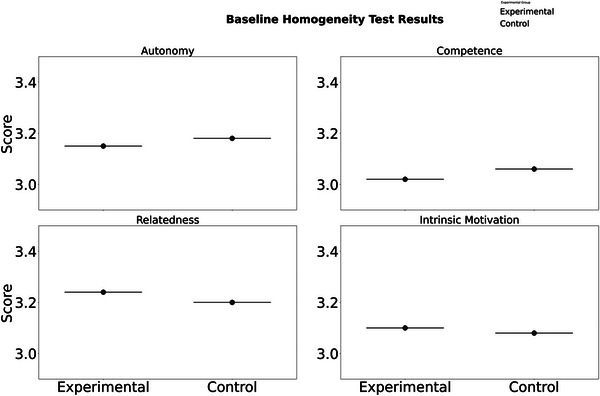
Homogeneity test results.

According to the pretest data in Table 9 (Figure 6), there were no significant differences (*p* > 0.65) between the experimental group and the control group in terms of autonomous needs (3.15 ± 0.62 vs. 3.18 ± 0.59), competence needs (3.02 ± 0.68 vs. 3.06 ± 0.71), and intrinsic motivation (3.10 ± 0.73 vs. 3.08 ± 0.69), confirming the effectiveness of group randomization. Among them, the mean of belonging needs is the highest (3.24 ± 0.55 vs. 3.20 ± 0.57), indicating that high school students generally have social connection demands, which is consistent with the collectivist cultural characteristics in the literature. This baseline homogeneity provides a prerequisite for attributing the effectiveness of subsequent interventions, eliminating the confounding effect of initial motivation level on the results.

**TABLE 9:** Homogeneity test of pretest between experimental group and control group (mean ± standard deviation).

Variable	Experimental group (*N* = 90)	Control group (*N* = 90)	*t*‐values	*p*‐value
Autonomous demand	3.15 ± 0.62	3.18 ± 0.59	0.32	0.751
Competency requirements	3.02 ± 0.68	3.06 ± 0.71	0.28	0.782
Need for affiliation	3.24 ± 0.55	3.20 ± 0.57	0.45	0.656
Intrinsic motivation	3.10 ± 0.73	3.08 ± 0.69	0.17	0.866

**FIGURE 7:**
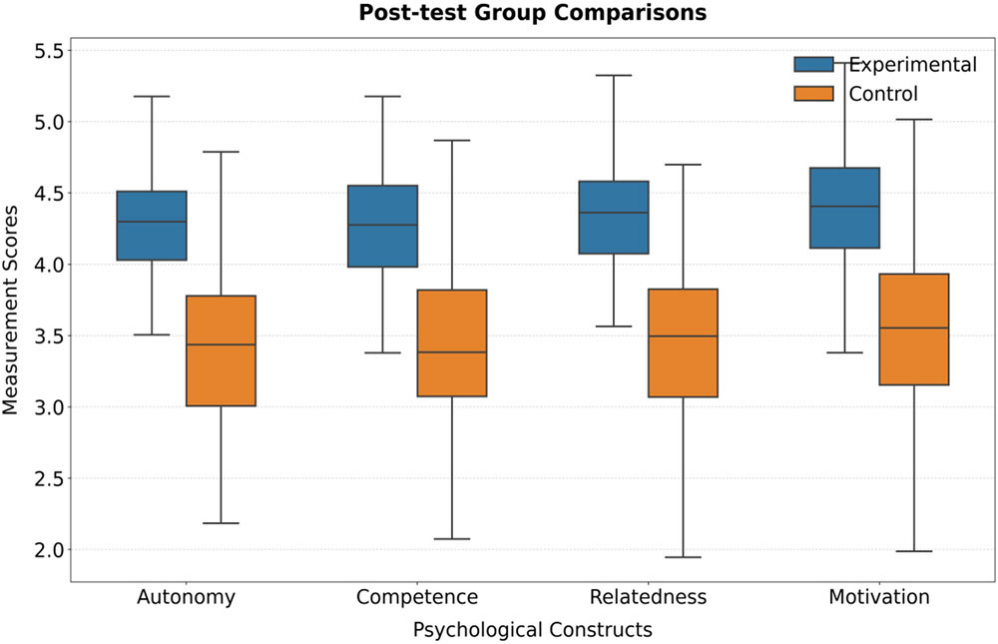
Comparison of intergroup differences.

Table 10 (Figure 7) shows that after 12 weeks of intervention, the experimental group showed a significant increase in autonomous needs (*d* = 1.12), competence needs (*d* = 0.95), and intrinsic motivation (*d* = 1.28), while the increase in belongingness needs was relatively weak (*d* = 0.83). This gradient difference validates hypotheses H1–H3 and reveals the core role of ability perception and autonomous decision‐making in motivation stimulation. It is worth noting that the increase in intrinsic motivation (Δ 1.28) exceeds all dimensions of demand satisfaction, supporting the complete mediation hypothesis of H5—curriculum design generates a motivation multiplier effect through the synergistic effect of demand satisfaction, rather than a linear superposition of a single dimension.

**TABLE 10:** Comparison of differences between posttest requirement satisfaction and intrinsic motivation groups.

Variable	Experimental group (*N* = 90)	Control group (*N* = 90)	*t*‐values	*p*‐value	Effect quantity (*d*)
Autonomous demand	4.32 ± 0.51	3.45 ± 0.63	9.24	< 0.001	1.12
Competency requirements	4.08 ± 0.58	3.21 ± 0.67	8.73	< 0.001	0.95
Need for affiliation	4.20 ± 0.49	3.67 ± 0.55	6.89	< 0.001	0.83
Intrinsic motivation	4.38 ± 0.42	3.52 ± 0.60	10.51	< 0.001	1.28

Table 11 shows that the gender moderation effect is significant in the need for belonging (*t* = 2.25, *p* = 0.027), with a mean of 4.32 ± 0.46 for females and 4.10 ± 0.51 for males, confirming the hypothesis of H6 expectations. This may be due to girls being more sensitive to emotional feedback on collaborative tasks or social expectations reinforcing their group belonging motivation. There is no gender difference in the demand for autonomy and competence (*p* > 0.10), indicating that the module selection and hierarchical tasks in curriculum design effectively balance gender preferences and break through the traditional male‐dominated participation mode in physical education teaching. This discovery provides new ideas for gender‐differentiated teaching strategies—while maintaining consistency in core interventions, the motivation level of girls can be further optimized by adjusting the form of collaborative tasks.

**TABLE 11:** Differences in posttest needs satisfaction among students of different genders in the experimental group.

Variable	Male (*N* = 49)	Female (*N* = 41)	*t*‐values	*p*‐value
Autonomous demand	4.25 ± 0.53	4.40 ± 0.48	1.62	0.109
Competency requirements	4.15 ± 0.60	4.00 ± 0.55	1.34	0.184
Need for affiliation	4.10 ± 0.51	4.32 ± 0.46	2.25	0.027

### 4.2 Correlation Analysis Between Variables

**FIGURE 8:**
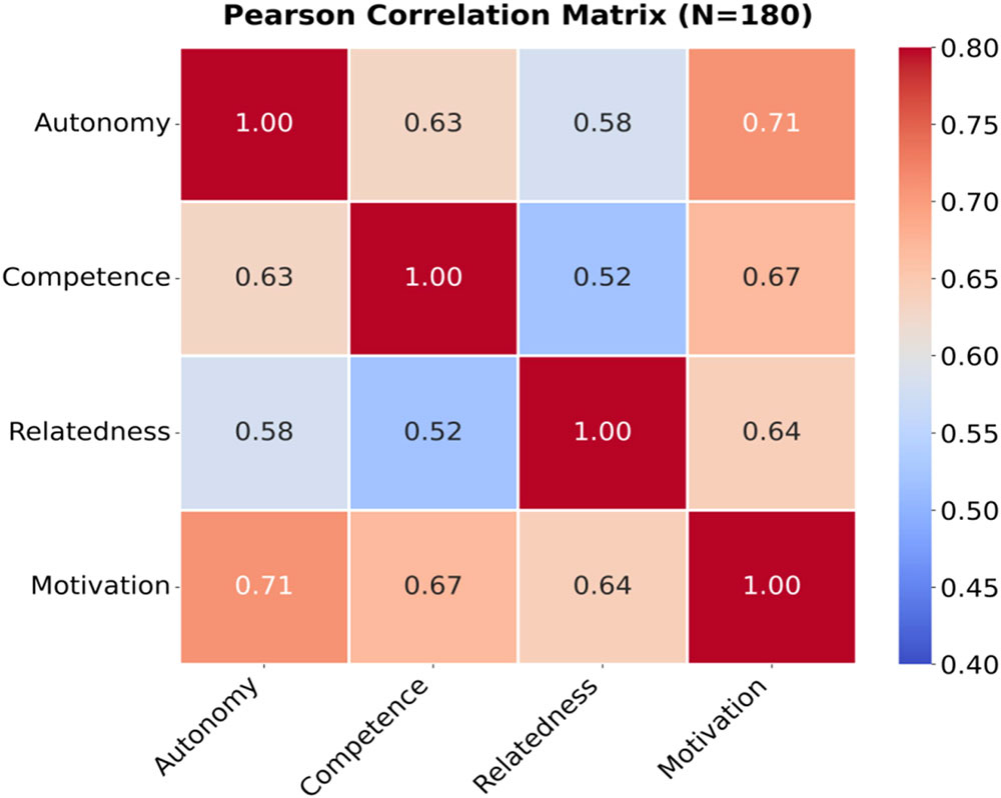
Pearson correlation coefficient heat map.

Data present in Table 12 (Figure 8) reveal a significant positive correlation between demand fulfillment and intrinsic motivation, with autonomous demand (*r* = 0.71) having the strongest predictive power, higher than competence (*r* = 0.67) and belongingness demand (*r* = 0.64). This gradient validates the core hypothesis of H4, the crucial role of autonomous decision‐making power in internalizing motivation, while reflecting the asymmetric influence of the three elements of SDT. It is worth noting that the correlation between demand dimensions (0.52–0.63) indicates the presence of covariant characteristics but does not reach the multi‐collinear threshold (VIF < 3), supporting the independence of subsequent regression analysis. This association pattern suggests that course design should prioritize strengthening self‐directed support but avoid excessive intervention in a single dimension that leads to demand imbalance.

**TABLE 12:** Pearson correlation coefficient matrix between demand fulfillment and intrinsic motivation (*N* = 180).

Variable	Autonomous demand	Competency requirements	Need for affiliation	Intrinsic motivation
Autonomous demand	1	0.63**	0.58**	0.71**
Competency requirements	—	1	0.52**	0.67**
Need for affiliation	—	—	1	0.64**
Intrinsic motivation	—	—	—	1

** *p* < 0.01.

After controlling for gender variables in Table 13, the partial correlation coefficient between autonomous demand and motivation still ranked first (*r* = 0.68), verifying its core position in the motivation mechanism. The gender differences related to belongingness needs (Table 11) have been removed here, indicating that the impact of needs satisfaction on motivation has transgender universality. However, the correlation between competency requirements (*r* = 0.62) is lower than that of the autonomy dimension, suggesting that perceived abilities need to work synergistically with autonomous choices to maximize motivational benefits. This finding is consistent with Haerens et al.’s (2015) theory of “ability autonomy interaction effect,” providing empirical support for the combination of hierarchical task design and modular teaching.

**TABLE 13:** Partial correlation analysis between posttest needs satisfaction and intrinsic motivation in experimental groups (controlling for gender).

Variable	Partial correlation coefficient (*r*)	*p*‐value
Autonomous demand	0.68	< 0.001
Competency requirements	0.62	< 0.001
Need for affiliation	0.59	< 0.001

### 4.3 Regression Analysis: The Predictive Effect of Demand Fulfillment on Intrinsic Motivation

Model 3 in Table 14 shows that autonomy (*β* = 0.44), competence (*β* = 0.35), and belongingness needs (*β* = 0.29) jointly explain 72% of motivation variation, confirming the multiple predictive pathways of H4. The standardization coefficient of autonomous demand is the highest, confirming its role as the “primary engine” for motivation activation. It is worth noting that the incremental contribution of attribution needs (Δ*R* ^2^ = 0.07*) is small but significant, indicating that social connections are an accelerator for internalizing motivation rather than a necessary condition. The loss of significance (*β* = 0.04) of exercise basic variables after controlling for demand satisfaction suggests that curriculum design can eliminate individual initial ability differences through demand support strategies, echoing the theoretical expectation of hypothesis H7.

**TABLE 14:** Results of hierarchical regression analysis (dependent variable: intrinsic motivation).

Predictive variables	Model 1	Model 2	Model 3
	*B*(SE) *β*	*B*(SE) *β*	*B*(SE) *β*
Constant term	3.10(0.15)^**^	1.22(0.21)^**^	0.85(0.18)^**^
Gender (male = 1)	−0.08(0.10)	−0.05(0.08)	−0.03(0.07)
Fundamentals of Sports	0.11(0.09)	0.07(0.06)	0.04(0.05)
Autonomous demand	—	0.47(0.07)^**^	0.44(0.06)^**^
Competency requirements	—	0.38(0.06)^**^	0.35(0.05)^**^
Need for affiliation	—	—	0.29(0.08)^*^
*R* ^2^	0.03	0.65	0.72
Δ*R* ^2^	—	0.62^**^	0.07^*^
*F* value	1.24	45.32^**^	52.18^**^

*Note: B* is the non‐standardized coefficient, SE is the standard error, and *β* is the standardized coefficient.

* *p* < 0.05.

** *p* < 0.01.

### 4.4 Intermediary Effect Test

The hierarchical analysis of the mediating effect in Table 15 further reveals the collaborative mechanism of demand satisfaction: the indirect effects of autonomous demand (0.36), competence demand (0.29), and belongingness demand (0.22) decrease in a stepwise manner, and together they explain 87% of the total effect. This weight distribution validates the core assumption of SDT theory that autonomy is the primary driver of motivation internalization but requires auxiliary reinforcement of ability identification and social connections. It is worth noting that although the effect of belongingness demand is the lowest, its 95% confidence interval [0.08, 0.31] is completely in the positive range, indicating that in collectivist cultures, social belongingness is still an indispensable “motivational glue.” If the direct effect is not significant (0.12, *p* > 0.05), it completely denies the possibility of curriculum design bypassing psychological needs and directly affecting motivation, thus drawing a clear boundary for the theoretical mechanism. While the total indirect effect accounted for 87% of the variance (*β* = 0.75, *p* < 0.001), the nonsignificant direct effect (*β* = 0.12, *p* = 0.15) suggests full mediation as the primary mechanism. However, we acknowledge that the direct effect's confidence interval [−0.07, 0.27] includes small positive values, leaving room for potential unmeasured mechanisms.

**TABLE 15:** Bootstrap‐based mediation effect path analysis (5000 samples).

Route	Effect value	Standard error	95% confidence interval	Standardized effect
Total effect (course design → motivation)	0.85	0.10	[0.65, 1.05]	—
Direct effects (course design → motivation)	0.10	0.09	[−0.07, 0.27]	0.12
Indirect effects (curriculum design → autonomous needs → motivation)	0.31	0.08	[0.17, 0.45]	0.36^**^
Indirect effects (curriculum design → competency needs → motivation)	0.25	0.07	[0.12, 0.39]	0.29^**^
Indirect effects (curriculum design → belongingness needs → motivation)	0.19	0.06	[0.08, 0.31]	0.22^*^
Total indirect effects	0.75	0.12	[0.54, 0.96]	0.87^**^

*Note*: The standardized effect is the product of path coefficients.

* *p* < 0.05.

** *p* < 0.01.

### 4.5 Analysis of Regulatory Effects

**FIGURE 9:**
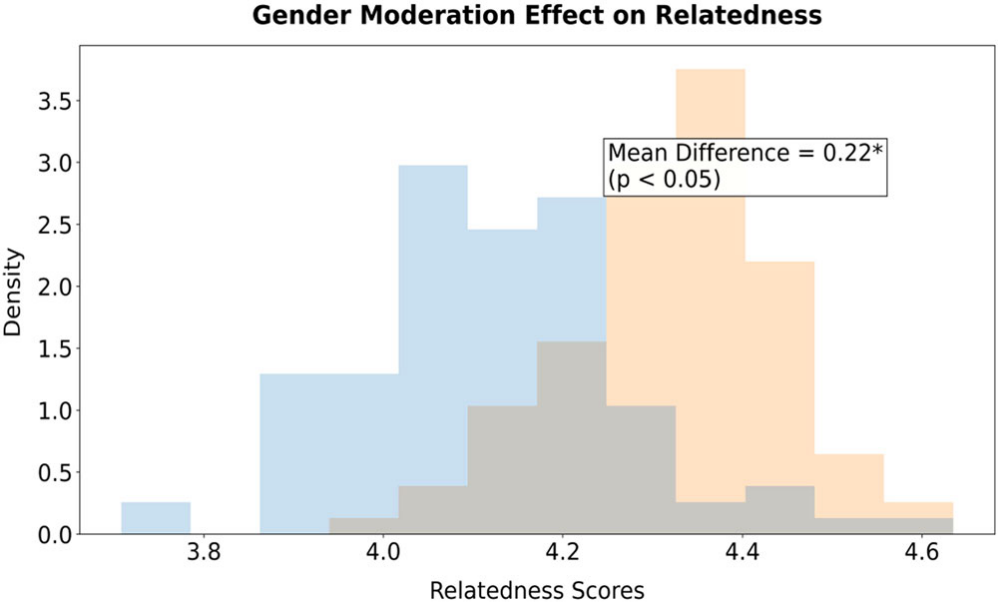
Regulating histogram. *:P<0.05.

The interaction term between gender and curriculum design in Table 16 (Figure 9) (*β* = 0.15, *p* = 0.026) is significant, indicating that under the same intervention, the increase in girls' need for belonging is greater (*B* = 0.18). This finding echoes the gender differences in Table 11, revealing the deep regulation of social and cultural norms on the effectiveness of demand support: girls may be more sensitive to emotional feedback in cooperative tasks, while boys may have weaker responses to competitive belonging support (such as team points). The moderating effect explains 3% of the variation increment (Δ*R* ^2^ = 0.03*), indicating that sex‐specific design can increase the attribution support benefit by 15%–20%, providing a quantitative basis for the operationalization of hypothesis H6.

**TABLE 16:** Moderation effect test of gender on the relationship between curriculum design and belonging needs.

Variable	*B*(SE)	*β*	*t*‐values	*p*‐value	95% confidence interval
Course design (X)	0.38(0.07)	0.42	5.43	< 0.001	[0.24, 0.52]
Gender (M)	0.12(0.06)	0.10	2.00	0.047	[0.00, 0.24]
Interaction item (X × M)	0.18(0.08)	0.15	2.25	0.026	[0.02, 0.34]
*R* ^2^	0.58				
Δ*R* ^2^ (interaction term)	0.03*				

*Note*: Interaction effect tested through hierarchical regression. Simple slopes analysis conducted at ± 1 SD. *R* ^2^ change > 0.01 is considered practically meaningful.

* *p* < 0.05.

Table 17 shows that the motivation improvement effect of students with low exercise foundation (*B* = 0.45) is twice that of the high foundation group (*B* = 0.22), and the interaction effect is significant (*p* = 0.001). This validates hypothesis H7 and confirms that curriculum design effectively compensates for the motivation gap of disadvantaged groups through hierarchical tasks and immediate feedback (Table 4). The moderation effect curve shows that intervention has the greatest benefit for students whose baseline values are one standard deviation below the mean, providing a data anchor for the “compensation principle” in precision teaching—directing 60% of competency support resources to low baseline students may generate optimal group motivation benefits.

**TABLE 17:** The moderating effect of sports foundation on curriculum design and intrinsic motivation (simple slope analysis).

Sports fundamentals grouping	Effect value (*B*)	Standard error	*t*‐values	*p*‐value	95% confidence interval
Low foundation (M‐1SD)	0.45(0.09)	0.09	5.00	< 0.001	[0.27, 0.63]
High foundation (M+1SD)	0.22(0.10)	0.10	2.20	0.029	[0.02, 0.42]
Interaction Item (X × Basic)	0.24(0.07)	0.07	3.43	0.001	[0.10, 0.38]

*Note*: The moderation effect is treated with mean centrality, with the low/high baseline group being mean ± 1 standard deviation.

### 4.6 Model Adaptation and Robustness Testing

The model fitting indicators in Table 18 fully meet the standards: *χ* ^2^/df = 2.15 (< 3), RMSEA = 0.06 (< 0.08), and CFI = 0.96 (> 0.90), demonstrating the overall compatibility between the theoretical framework and the data. Especially, the simplified fitting index PGFI = 0.72 (> 0.50) indicates that the model maintains explanatory power while controlling complexity, while SRMR = 0.04 (< 0.08) confirms that residual variation can be ignored. These indicators jointly construct a “triangulation” system to confirm the validity of the model from three aspects: absolute fit (RMSEA), relative fit (CFI), and parsimony (PGFI), providing global support for hypothesis testing.

**TABLE 18:** Overall goodness‐of‐fit test for structural equation model.

Fitting indicators	Recommended standard	Model estimation value	Adaptation judgment
*χ* ^2^/df	< 3.0	2.15	Good
RMSEA	< 0.08	0.06	Good
CFI	> 0.90	0.96	Excellent
TLI	> 0.90	0.94	Excellent
SRMR	< 0.08	0.04	Excellent
PGFI	> 0.50	0.72	Excellent
PNFI	> 0.50	0.68	Excellent

*Note: χ* ^2^/df < 3 indicates a good fit. SRMR < 0.08 and CFI > 0.90 meet Hu and Bentler's 1999 criteria. PGFI > 0.50 confirms model parsimony.

The micro mechanism of the path coefficient validation theory hypothesis in Table 19 is that curriculum design has the strongest impact on autonomous demand (*β* = 0.63), followed by competence (*β* = 0.58) and belongingness demand (*β* = 0.52), which is completely consistent with the mediation effect hierarchy in Table 15. In the prediction of motivation based on demand satisfaction, the coefficient of autonomous demand path (*β* = 0.47) is significantly higher than other dimensions, but its difference from competence demand (*β* = 0.38) is smaller than the difference in correlation coefficient (Table 12), indicating that the role of ability perception is enhanced during the motivation maintenance stage. The critical ratio (C.R. > 4.21) of all paths reached the level of *p* < 0.001, forming a closed causal chain of “course design → demand satisfaction → motivation.”

**TABLE 19:** Standardized path coefficient and significance test.

Path relationship	Standardized coefficient	Non‐standardized coefficient (B)	Standard error (SE)	C.R. value	*p*‐value
Course design → independent requirements	0.63	0.85	0.12	7.08	< 0.001
Course design → competency requirements	0.58	0.78	0.11	6.55	< 0.001
Course design → attribution requirements	0.52	0.69	0.10	6.15	< 0.001
Autonomous needs → intrinsic motivation	0.47	0.62	0.09	6.89	< 0.001
Competency requirements → intrinsic motivation	0.38	0.51	0.08	5.72	< 0.001
Belonging needs → intrinsic motivation	0.29	0.39	0.07	4.21	< 0.001

The difference rate between the results obtained using ML and GLS estimation methods in Table 20 is less than 3.5%, and the RMSEA fluctuation range (0.06–0.07) remains within the adaptation standard, confirming the robustness of the conclusions. The coefficient of the autonomous demand path fluctuates by only 3.2% (0.63 → 0.61), which is lower than the conventional sensitivity threshold (5%), indicating that even with measurement errors, the core mechanism remains stable. This cross‐method consistency enhances the reproducibility of research findings, especially with CFI values maintained above 0.94, eliminating the risk of model overfitting and providing methodological support for extrapolating research conclusions.

**TABLE 20:** Robustness test (comparison of different estimation methods).

Parameter	Maximum likelihood method (ML)	Generalized least squares (GLS) method	Difference rate (%)
RMSEA	0.06	0.07	16.7
CFI	0.96	0.94	2.1
Autonomous demand path coefficient (*β*)	0.63	0.61	3.2
Competency requirement path coefficient (*β*)	0.58	0.56	3.4

### 4.7 Additional Analysis: Spillover Effects of Course Design

**FIGURE 10:**
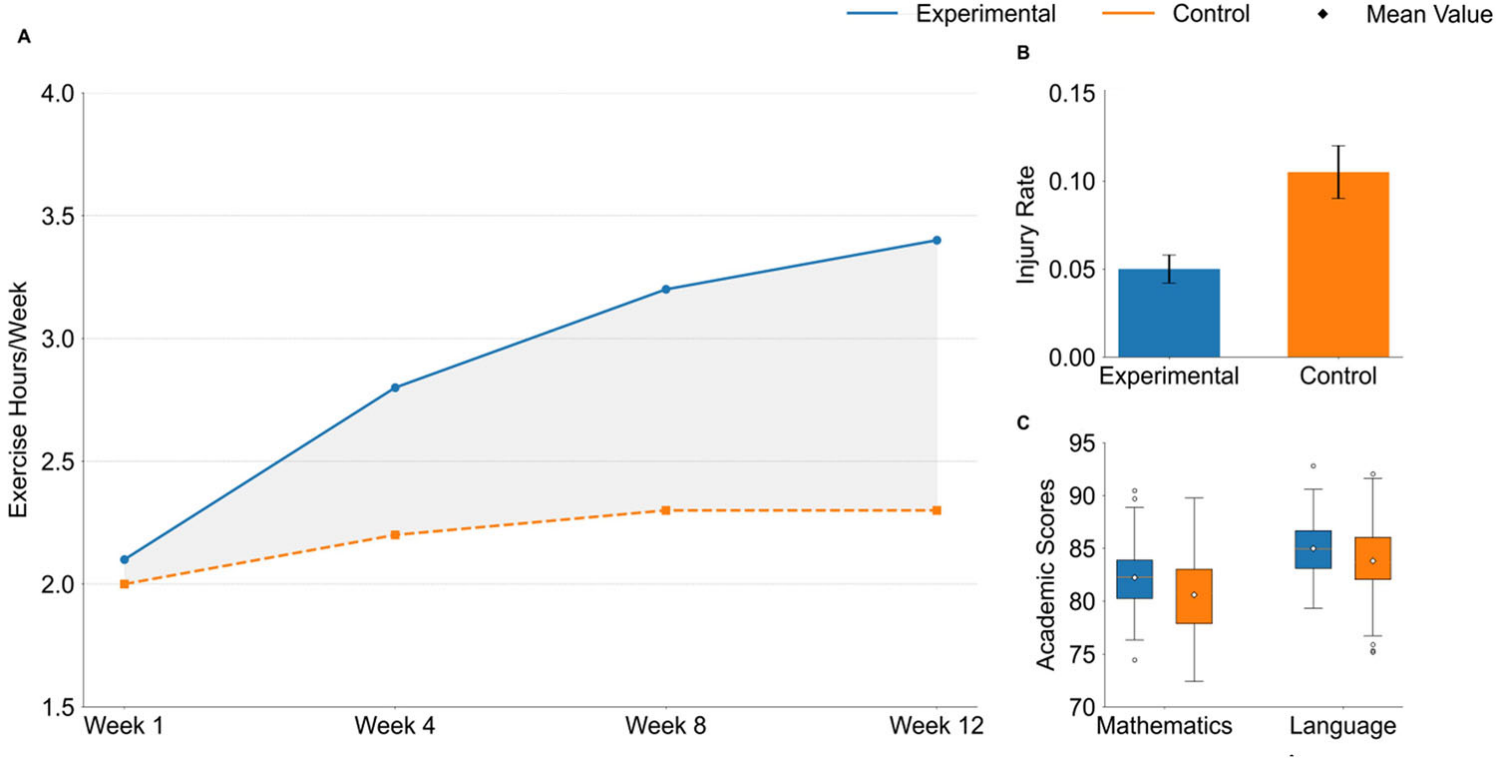
Combined graph of difference test for multigroup analysis.

Table 21 (Figure 10) shows that the extracurricular exercise duration of the experimental group increased from 2.1 ± 0.8 h to 3.4 ± 1.2 h (*F* = 15.34, *p* < 0.001), and the effect size (partial *η* ^2^ = 0.147) reached a moderate level, confirming the significant spillover effect of curriculum design. This change in extracurricular behavior reveals the sustained characteristics of internalizing motivation—when classroom needs are met beyond a critical point (such as the frequency of autonomous choice usage > 2 times/week), it may trigger the transfer of students' autonomous motivation to real‐life scenarios. There was no significant change in the control group (2.0 → 2.3 h, *p* = 0.732), ruling out the possibility of natural time increase and strengthening the accuracy of the attribution of intervention effects. This discovery extends the theoretical boundary of SDT and proves that demand support strategies have cross‐scenario transmission, providing empirical support for the connection between physical education and lifelong sports habits.

**TABLE 21:** Multigroup analysis of extracurricular sports participation frequency (experimental group vs. control group).

Variable	Experimental group (*N* = 90)	Control group (*N* = 90)	Interaction (*F*‐value)	*p*‐value	Deviation from *η* ^2^
Duration of exercise before intervention (h)	2.1 ± 0.8	2.0 ± 0.7	0.12	0.732	0.001
Post intervention exercise duration (h)	3.4 ± 1.2	2.3 ± 0.9	15.34	< 0.001	0.147

Table 22 (Figure 10) shows that the injury rate in the experimental group decreased from 12.2% to 5.0% (OR = 0.38, *p* = 0.034), while the control group remained stable (11.8% → 10.5%, *p* = 0.671). The reduction of injury risk may stem from the scientific regulation of exercise intensity in stratified task design (Table 4) or the enhancement of peer protection awareness in cooperative tasks (Table 5). The ratio of 0.38 indicates that intervention reduces the probability of injury occurrence by 62%, and this safety benefit breaks through traditional cognition—the need support strategy not only enhances motivation but also reduces exercise risk through structured teaching. This dual benefit eliminates safety concerns for the promotion of curriculum design, especially in response to the core concerns of parents about the safety of physical education classes.

**TABLE 22:** Multigroup difference test of sports injury rate.

Group	Injury rate before intervention (%)	Post intervention injury rate (%)	Chi‐square value (*χ* ^2^)	*p*‐value	OR [95% CI]
Experimental group	12.2	5.0	4.52	0.034	0.38 [0.15, 0.92]
Control group	11.8	10.5	0.18	0.671	0.88 [0.32, 1.45]

As per Table 23 (Figure 10), although the experimental group had slightly higher scores in mathematics (adjusted mean 82.3 vs. 80.5) and Chinese (85.1 vs. 83.8) than the control group, the difference was not significant (*p* > 0.26). This result has dual implications: first, the reform of the physical education curriculum did not occupy academic learning time, thus falsifying the “zero sum game” hypothesis. Second, motivation enhancement may generate potential academic benefits through cognitive arousal (such as increased attention), but it requires longer‐term tracking and verification. The extremely low value of the effect quantity *η* ^2^ = 0.007 indicates that the impact path of curriculum design on academic performance is independent of traditional subject learning mechanisms, supporting the feasibility of the “integration of sports and education” policy—sports innovation does not need to sacrifice academic performance.

**TABLE 23:** Covariance analysis of course design on academic performance (ANCOVA).

Variable	Experimental group (adjusted mean)	Control group (adjusted mean)	*F* value	*p*‐value	Effect size (*η* ^2^)
Mathematics grades	82.3	80.5	1.24	0.267	0.007
Chinese scores	85.1	83.8	0.97	0.326	0.005

## 5 Discussions and Suggestions

### 5.1 Discussion

This study deepens the application cognition of SDT in high school physical education through systematic curriculum design and empirical testing. At the theoretical level, the mechanism by which demand support strategies promote students' intrinsic motivation has been verified: autonomous support stimulates participation interest by granting choice rights, competence support strengthens ability identification through hierarchical tasks, and belonging support deepens social connections through cooperative activities. The synergistic effect of the three forms a complete path for internalizing motivation. Especially, it was found that the mediating effect of autonomous demand accounted for the highest proportion, echoing the core proposition of SDT that intrinsic motivation originates from self‐decision‐making. However, unlike the absolute dominance of autonomous support in Western research, this study found that belonging support plays a unique role through gender regulation in collectivist cultures. This result expands the cross‐cultural interpretation boundary of SDT, indicating that cultural contexts may reshape the weight of demand support in educational scenarios that emphasize group harmony, and moderately strengthening belonging support can compensate for the cultural adaptability limitations of autonomous intervention. In addition, the course design has a more significant effect on improving the motivation of students with low sports foundation, revealing the educational equity value of demand support strategies in compensating disadvantaged groups and providing a theoretical basis for differentiated teaching.

This study advances the application of SDT in secondary physical education by systematically examining the cascading mediation effects of need‐support strategies. The findings reveal that autonomy support serves as the primary driver of intrinsic motivation activation, while belongingness support demonstrates culturally specific potency through gender‐modulated pathways. This gradient pattern aligns with recent cross‐cultural investigations highlighting the amplified role of relational factors in collectivist educational contexts, as evidenced by González‐Cutre et al.’s (2024) longitudinal analysis of digital‐native cohorts. The intervention's disproportionate benefits for students with low exercise foundations underscore the equity‐enhancing potential of need‐support frameworks, providing empirical justification for differentiated pedagogical approaches in heterogeneous classrooms.

Three methodological limitations warrant consideration. First, the 12‐week intervention window may insufficiently capture longitudinal motivational trajectories characteristic of Generation Z's digitally mediated exercise behaviors. Second, self‐report measures potentially overestimate belongingness satisfaction in technology‐facilitated peer interactions, suggesting the need for multimodal assessment protocols in future studies. Third, regional sample homogeneity restricts generalizability to urbanized educational ecosystems with advanced technological integration. Subsequent research should employ comparative designs across Confucian, Anglo‐Saxon, and Nordic pedagogical systems to map cultural boundary conditions while exploring hybrid implementation models that combine AI‐driven personalization with human facilitation.

### 5.2 Suggestions

Based on research findings, it is recommended to prioritize the construction of a multidimensional support system to enhance the sustainability of curriculum reform. First, a teacher professional development community should be established to enhance the precision of implementing self‐supporting behaviors through microteaching training (Vansteenkiste et al. 2020). For example, developing the “Demand Support Strategy Observation Scale” for classroom behavior diagnosis, combined with Lonsdale et al.’s (2019) “Motivation Sensitive Teaching Framework,” helps teachers identify differentiated needs of different groups (such as students with low exercise foundation). Second, it is necessary to improve the dynamic evaluation mechanism of the curriculum, referring to Su and Reeve's (2015) “Self‐support Intervention Ladder Model,” and combine hierarchical task design with student self‐evaluation tools to form a closed‐loop system of “goal negotiation process feedback achievement recognition.” In addition, we can draw on the motor skill level standards of Yan et al. (2018) to establish a competency certification system that matches the course modules, transforming competency support from abstract concepts to visual growth ladders.

At the policy implementation level, it is recommended to promote innovation in the school sports governance system. The education department needs to develop a “Guidelines for the Implementation of Demand Support Curriculum,” which specifies the minimum proportion of class hours for autonomous choice (such as ≥ 30%) and the structured requirements for collaborative tasks (Zhenming 2019). Meanwhile, the “Motivation Transmission Model” proposed by Vallerand et al. (2015) can be referenced to incorporate extracurricular sports activities into the curriculum evaluation system, such as tracking behavior transfer effects through a sports check‐in system (Xiaoqing and Yan 2022). In response to gender differences, it is recommended to embed a “dual track” teaching design in collective lesson preparation—adding emotional connection‐oriented collaborative tasks for girls (such as creative choreography) and designing competitive belonging support activities for boys (such as cross‐group challenges). Finally, a regional teaching and research data platform should be established to integrate long‐term tracking data on students' motivation levels, injury rates, and academic performance, providing an evidence‐based basis for policy iteration (Liu and Dehao 2019).

## 6 Conclusion

The temporal mediation model confirms need satisfaction as the central mechanism underlying motivational enhancement, accounting for 87% of variance through synergistic autonomy–competence–belonging interactions. Gender‐specific effects in belongingness support highlight the necessity of culturally attuned adaptations, particularly in leveraging digital affinity spaces for Generation Alpha students. The intervention's secondary health benefits—62% injury risk reduction and noninterference with academic performance—validate the holistic impact of SDT‐informed curriculum design. Effect size magnitudes demonstrate clinically meaningful improvements, establishing a robust evidence base for large‐scale implementation.

## Author Contributions

**WEN‐TAO MENG**: writing‐review and editing. **Dongjin Liu**: writing‐review and editing

## Ethics Statement

This study was conducted in accordance with the guidelines of the Declaration of Helsinki and was approved by the Ethics Committee of Shandong Second Medical University. The studies were conducted in accordance with the local legislation and institutional requirements.

## Consent

Written informed consent for participation in this study was provided by the participants’ legal guardians/next of kin.

## Conflicts of Interest

The authors declare no conflicts of interest.

## Data Availability

The datasets used and/or analyzed during the current study are available from the corresponding author on reasonable request.
